# ^31^P MR Spectroscopy and Computational Modeling Identify a Direct Relation between Pi Content of an Alkaline Compartment in Resting Muscle and Phosphocreatine Resynthesis Kinetics in Active Muscle in Humans

**DOI:** 10.1371/journal.pone.0076628

**Published:** 2013-09-30

**Authors:** Joep W. M. van Oorschot, Joep P. J. Schmitz, Andrew Webb, Klaas Nicolay, Jeroen A. L. Jeneson, Hermien E. Kan

**Affiliations:** 1 Department of Radiology, University Medical Center Utrecht, Utrecht, The Netherlands; 2 Systems Bioinformatics, Amsterdam Institute for Molecules, Medicines and Systems (AIMMS), VU University Amsterdam, Amsterdam, The Netherlands; 3 Computational Biology group, Department of Biomedical Engineering, Eindhoven University of Technology, Eindhoven, The Netherlands; 4 CJ Gorter Center for High field MRI, Department of Radiology, Leiden University Medical Center, Leiden, The Netherlands; 5 Biomedical NMR, Department of Biomedical Engineering, Eindhoven University of Technology, Eindhoven, The Netherlands; 6 Laboratory for Liver, Digestive and Metabolic Disease, University Medical Center Groningen, Groningen, The Netherlands; West Virginia University School of Medicine, United States of America

## Abstract

The assessment of mitochondrial properties in skeletal muscle is important in clinical research, for instance in the study of diabetes. The gold standard to measure mitochondrial capacity non-invasively is the phosphocreatine (PCr) recovery rate after exercise, measured by ^31^P Magnetic Resonance spectroscopy (^31^P MRS). Here, we sought to expand the evidence base for an alternative method to assess mitochondrial properties which uses ^31^P MRS measurement of the Pi content of an alkaline compartment attributed to mitochondria (Pi_2_; as opposed to cytosolic Pi (Pi_1_)) in resting muscle at high magnetic field. Specifically, the PCr recovery rate in human quadriceps muscle was compared with the signal intensity of the Pi_2_ peak in subjects with varying mitochondrial content of the quadriceps muscle as a result of athletic training, and the results were entered into a mechanistic computational model of mitochondrial metabolism in muscle to test if the empirical relation between Pi_2_/Pi_1_ ratio and the PCr recovery was consistent with theory. Localized ^31^P spectra were obtained at 7T from resting vastus lateralis muscle to measure the intensity of the Pi_2_ peak. In the endurance trained athletes a Pi_2_/Pi_1_ ratio of 0.07 ± 0.01 was found, compared to a significantly lower (p<0.05) Pi_2_/Pi_1_ ratio of 0.03 ± 0.01 in the normally active group. Next, PCr recovery kinetics after in magnet bicycle exercise were measured at 1.5T. For the endurance trained athletes, a time constant τ_PCr_ 12 ± 3 s was found, compared to 24 ± 5s in normally active subjects. Without any parameter optimization the computational model prediction matched the experimental data well (*r* ^2^ of 0.75). Taken together, these results suggest that the Pi_2_ resonance in resting human skeletal muscle observed at 7T provides a quantitative MR-based functional measure of mitochondrial density.

## Introduction

Non-invasive assessment of mitochondrial properties in human tissues including skeletal muscle is important in clinical research, for instance in diabetes, and sports medicine [1]. In vivo ^31^P MR spectroscopy has been widely used for this assessment [2]. Specifically, the rate of phosphocreatine (PCr) recovery after exercise is commonly used as an index for mitochondrial capacity [3]. This method has, however, some disadvantages. First of all, it requires in-magnet exercise and therefore a complex setup. In addition, some patients are not able to perform exercise inside the magnet. Another drawback is that this method provides only an indirect readout of mitochondrial capacity. PCr recovery is not only limited by mitochondrial density and functionality, but also by perfusion of the muscle tissue [4], and the intensity of the exercise [5].

An alternative ^31^P MRS technique to assess mitochondrial properties non-invasively is offered by magnetization transfer measurements, in which the oxidative ATP synthesis flux is measured by saturation of the γ-ATP peak resonance at 2.5 ppm [6]. The advantage of this method over the PCr recovery assay is that it can be performed in resting muscle. However, the flux obtained from a ^31^P saturation transfer experiment is dominated by the glycolytic exchange flux instead of the mitochondrial ATP synthesis flux [7]. As a result, the readout of mitochondrial properties using magnetization transfer is not straightforward. A more direct and fast *in vivo* measurement of mitochondrial properties under resting conditions would provide a major advance compared to these current methods.

In previous work at a magnetic field strength of 7 Tesla [8] our group observed a peak 0.4 ppm downfield from the cytosolic Pi resonance (Pi_1_) in resting human skeletal muscle. Based on the chemical shift value, the T_1_ characteristics, and the difference in intensity of the second peak between the soleus and tibialis anterior muscles, this signal was putatively attributed to the Pi pool inside the mitochondrial matrix (Pi_2_) [8]. If confirmed, this signal could provide a new biomarker for mitochondrial properties in muscle that may be assayed in subjects at rest. In this paper, we further investigated if the Pi_2_ signal can provide information about mitochondrial properties. Specifically, the hypothesis was tested that the amplitude of the Pi_2_ signal in resting muscle is a good indicator of mitochondrial density. To test this hypothesis, we conducted static and dynamic in vivo ^31^P MRS measurements at 7T and 1.5T, respectively, in quadriceps muscle of normally-active subjects and trained athletes and tested if the relation between Pi_2_ signal intensity of resting muscle and the rate of PCr recovery following exercise followed the theoretical relation between mitochondrial Pi content and mitochondrial density derived from a computational model of oxidative metabolism in muscle [9].

## Experimental

### Subjects

The study was conducted in ten healthy volunteers (age range 20-27 years). Five subjects were highly trained endurance runners (exercise 6-9 times/week, 1-1.5 hour per training) (ATH). The other five subjects were reasonably physical active (running/cycling 1-2 times/week, 1 hour per training) (REG). Written informed consent was obtained from all participants, and this study was approved by the local Medical Ethics Committee of the Leiden University Medical Center.

### Static ^31^P MRS measurement at 7T

^31^P NMR data from resting skeletal muscle were acquired on a 7 Tesla Philips Achieva scanner (Philips Healthcare, Best, The Netherlands). Subjects were placed feet first in the magnet in a supine position. A custom-built transmit and receive double-tuned ^1^H and ^31^P coil setup, with square coils for ^31^P (10 cm) and ^1^H (12 cm), was placed on top of the vastus lateralis muscle of the right upper leg. A B_0_ map was acquired for the image based shimming algorithm [10]. Shimming was performed on a manually drawn region of interest in the lateralis muscle. ^31^P spectra were obtained using 2D chemical shift imaging (CSI) with a field of view (FOV) of 160x160 mm; matrix size 8x8; Hamming weighted acquisition with 32 averages at the center k-lines. Slice thickness was determined by the coil size (10 cm). Adiabatic half passage 90 degrees RF pulses of 3.3 ms duration were applied with the transmitter frequency set at 5.0 ppm downfield from the PCr peak. The repetition time was set to 1680 ms, resulting in a total measurement time for the 2D CSI of 20 minutes.

### Dynamic ^31^P MRS measurements at 1.5T

Within one week after the 7 Tesla studies, PCr recovery data were acquired from all volunteers on the 1.5 Tesla system (Philips Healthcare, Best, The Netherlands), since no in-magnet exercise setup is available for the 7 Tesla scanner. A custom-built transmit and receive double-tuned ^1^H and ^31^P coil setup with circular coils for ^31^P (5 cm) and ^1^H (6 cm) was used, interfaced to a Bruker Biospin console. Exercise was performed using a MR-compatible bicycle ergometer for in-magnet exercise [11]. ^31^P spectra were obtained with surface coil localization on the right vastus lateralis. To ensure similar coil placement in both the 7T and 1.5T scans, coil positioning was performed for all measurements by the same person. A pulse-acquire sequence was applied with adiabatic half passage pulses of 2 ms duration, with a repetition time of 3s. Two free induction decays (FIDs) were averaged per spectrum, resulting in a time resolution of 6 seconds. Before exercise, a fully relaxed rest spectrum was acquired (8 averages), with a TR of 30s. A light sensor was used to gate spectrometer data acquisition during cycling. Cycling was performed with a constant speed of 80 rotations per minute, indicated by a metronome. Exercise intensity was increased gradually, by adding weights onto the brake of the bicycle ergometer. Because PCr and Pi recovery are sensitive to cellular pH [5], we aimed for the same relative end-exercise intensity in each subject. Therefore the PCr level was monitored realtime, and exercise was stopped when a PCr depletion of 50% was reached, resulting in an end-exercise pH higher than 6.8 in all subjects. The total exercise time was 5 minutes on average. Directly after exercise, recovery was measured for 10 minutes.

### Data processing

**Figure 1:**
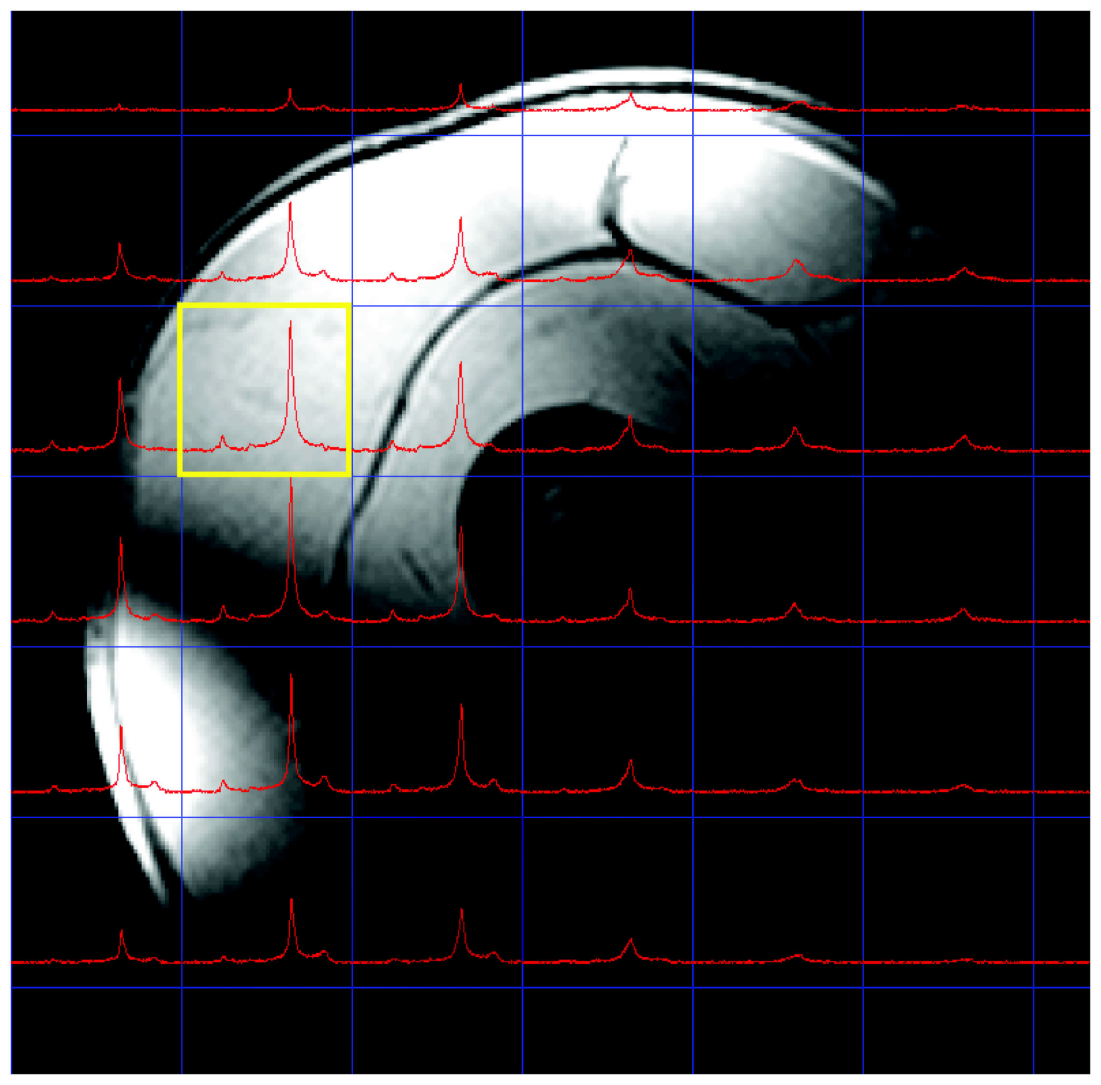
MRI image with CSI dataset. T_1_ weighted gradient echo image with the measured 2D CSI dataset. The selected voxel in the vastus lateralis muscle with the related ^31^P spectrum is shown in yellow.

The CSI dataset was visualized using 3DiCSI software, and a voxel was selected in the right lateralis muscle, located completely inside the muscle and remote from large visible blood vessels (Figure 1). The free induction decay was analyzed using the jMRUI software package. Peak areas for the two Pi signals from the 7T data and Pi, PCr and ATP signals from the 1.5T data were obtained by fitting Lorentzian line shapes and correcting for partial saturation effects [12,13]. Correction was applied with a T_1_ of 1.4 seconds for Pi_2_, and 4.3 seconds for Pi_1_ [8]. The line width of the Pi_2_ peak was constrained to the line width and phase of the Pi_1_ peak, to ensure a good fit for the Pi_2_ peak.

Using a least squares method the PCr recovery curve was fitted to a mono-exponential model, to obtain time constant τ_PCr_ [14]. The peak areas for ATP and Pi were obtained for the fully relaxed ^31^P spectra obtained at 1.5 T, and the Pi/ATP ratio was calculated.

### Computational Modeling

A detailed biophysical model of cardiac mitochondrial ATP metabolism proposed by Beard and coworkers [15], and adapted for skeletal muscle by Schmitz et al. [9,16] was applied to test if the empirical relation between Pi_2_/Pi_1_ and rate of PCr recovery was consistent with the theoretically expected relation. For a detailed description of the model we refer to [9]. In brief, the model comprises three cellular compartments (mitochondrial matrix, mitochondrial inter membrane space and cytoplasm). The model was developed by integration of mechanistic rate equations representing the processes of mitochondrial oxidative phosphorylation, adenine nucleotide transport across the mitochondrial membranes, cytoplasmic ATP buffering and hydrolysis. The accuracy of the model was previously tested by comparison of model predictions and data obtained from isolated mitochondria and ^31^P MRS observed metabolite dynamics in human and rodent muscle [9,16]. Model predictions were performed using Matlab version 7.14.

The model was applied to predict the PCr recovery time constant (τ_PCr_) for various mitochondrial volume fractions (ranging from 0.01 to 0.15 [ml mito/ml cell]). Each simulation was performed in three consecutive steps. First, a model initialization step was performed in which the model ATP hydrolysis rate was incrementally increased until a steady state PCr depletion level of 50% was achieved (similar to the experimental data). Next, cytoplasmic pH was set at 6.9, consistent with the end exercise values and ATP hydrolysis rate was decreased to resting values (0.01 mM/s [9]) in order to simulate 600s of recovery period. Finally, the time constant of PCr recovery (τ_PCr_) was derived from the predicted PCr recovery dynamics by fitting of a mono-exponential function.

For each simulation the corresponding Pi_2_/Pi_1_ ratio was calculated according to the following equation

Pi_2_/Pi_1_ = V_mito_ * [Pi_rest_]_mitoMatrix_ / [Pi_rest_]_cytoplasm_

where: V_mito_ is the mitochondrial volume fraction, [Pi_rest_]_mitoMatrix_ is the inorganic phosphate concentration in the mitochondrial matrix at rest and [Pi_rest_]_cytoplasm_ is the inorganic phosphate concentration in the cytoplasm. The [Pi_rest_]_mitoMatrix_ / [Pi_rest_]_cytoplasm_ ratio was predicted by the model ([Pi_rest_]_mitoMatrix_ / [Pi_rest_]_cytoplasm_ = 0.92).

### Statistics

Differences in pH, Pi_2_/Pi_1_, Pi/ATP and τ_PCr_ between the groups, trained athletes and healthy controls, were compared with a t-test and considered significant at *P*<0.05. The quality of the match between model predictions and experimental data was quantified by calculation of the coefficient of determination (R^2^).

## Results

### Static ^31^P MRS measurements at 7T

**Figure 2:**
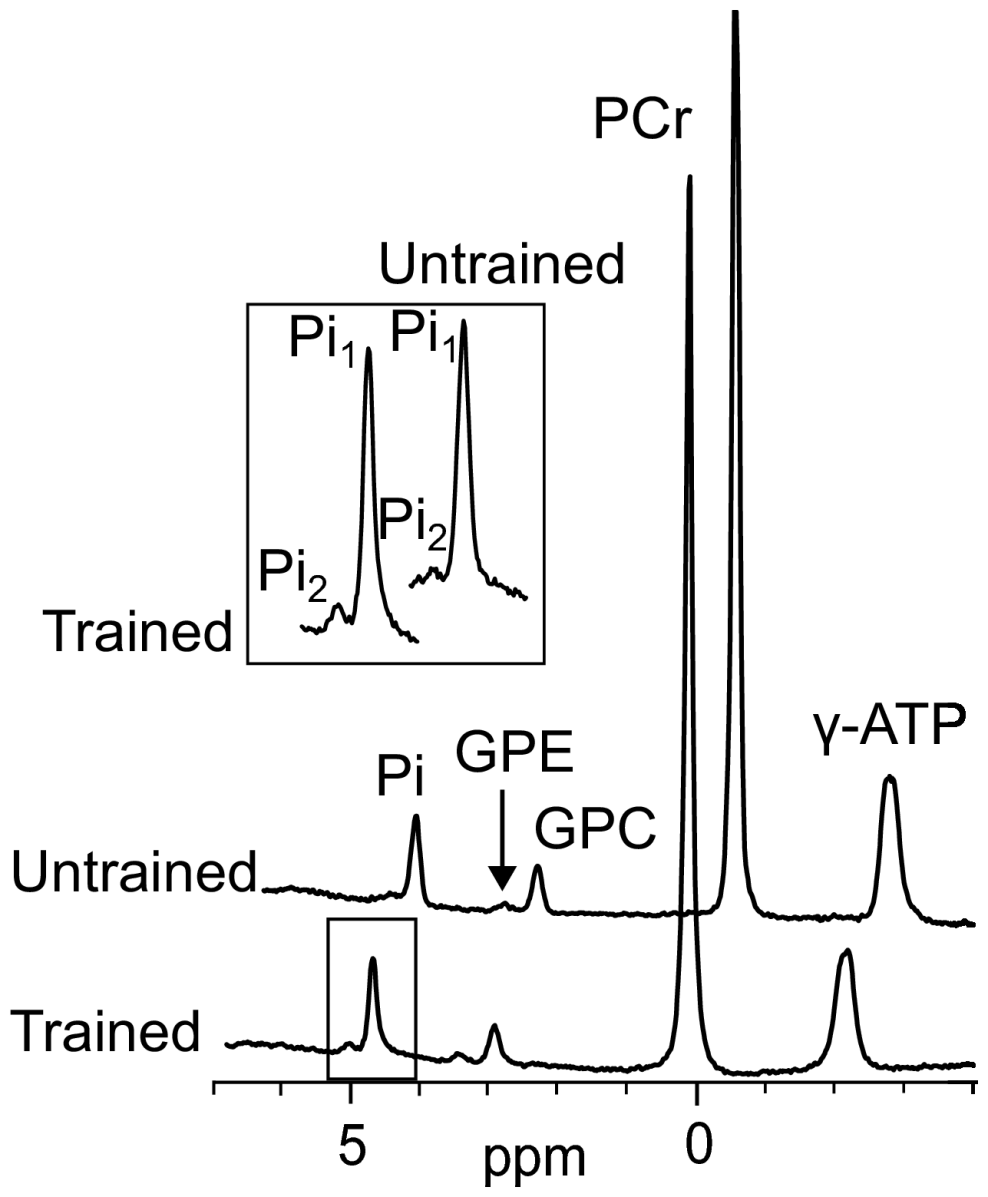
^31^P spectra from a trained and an untrained subject. ^31^P spectra from a trained and an untrained subject, after a 3 Hz Lorentzian window function was applied. The Pi_2_ intensity is higher in the trained subject. Spectra are scaled to the PCr resonance peak. Peaks visible: two signals for inorganic phosphate (Pi_1_ and Pi_2_), glycerol phosphocholine (GPC), glycerol phosphoethanolamine (GPE), phosphocreatine(PCr), γ-adenosine triphosphate (γ-ATP). The inset shown the two signals for inorganic phosphate (Pi_1_ and Pi_2_) in the trained and untrained subjects in more detail.

**Figure 3:**
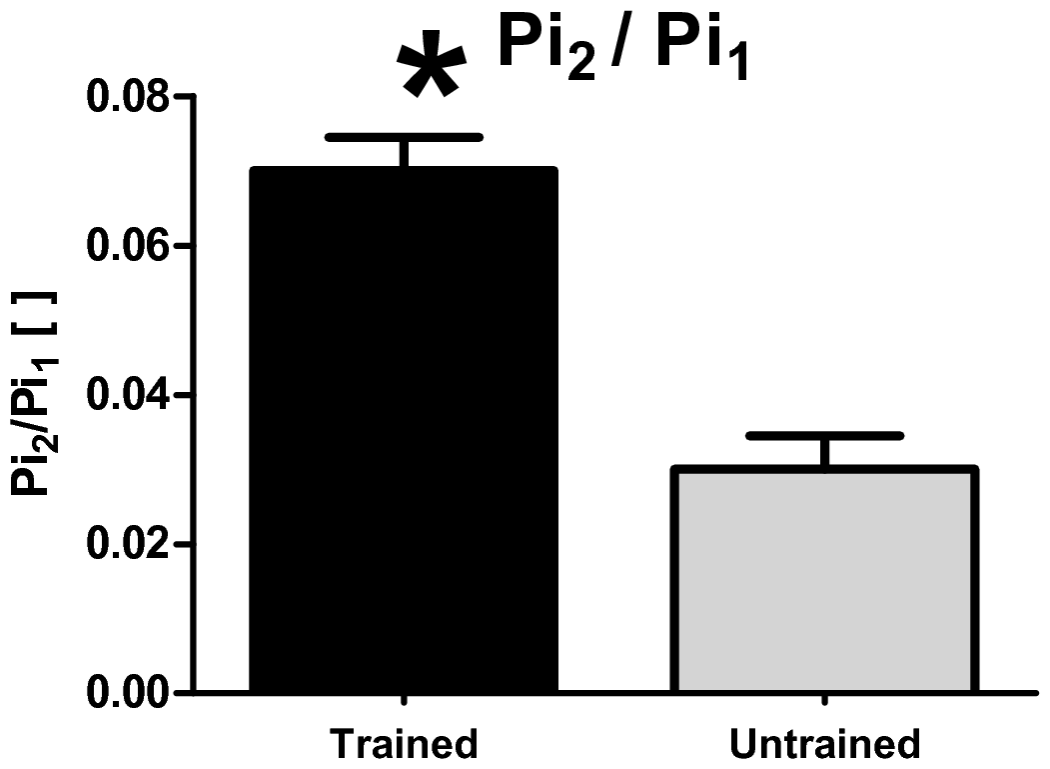
Bar plot Pi_2_/Pi_1_ ratio trained/untrained. Bar plot of Pi_2_/Pi_1_ ratio with a significant higher Pi_2_/Pi_1_ in the endurance trained athletes (0.07 ± 0.01) compared to the normal physical active group (0.03 ± 0.01) (P < 0.05).

A typical 7T spectrum is shown in Figure 2. A peak at 0.4 ppm downfield from the cytosolic Pi peak was detected in all subjects, indicating an alkaline pH compartment (Figure 2). In the endurance trained athletes a Pi_2_/Pi_1_ ratio of 0.07 ± 0.01 was found. In the normally-active group the Pi_2_/Pi_1_ ratio was significantly lower at 0.03 ± 0.01 (Figure 3).

### Dynamic^31^PMRS measurements at 1.5T

**Figure 4:**
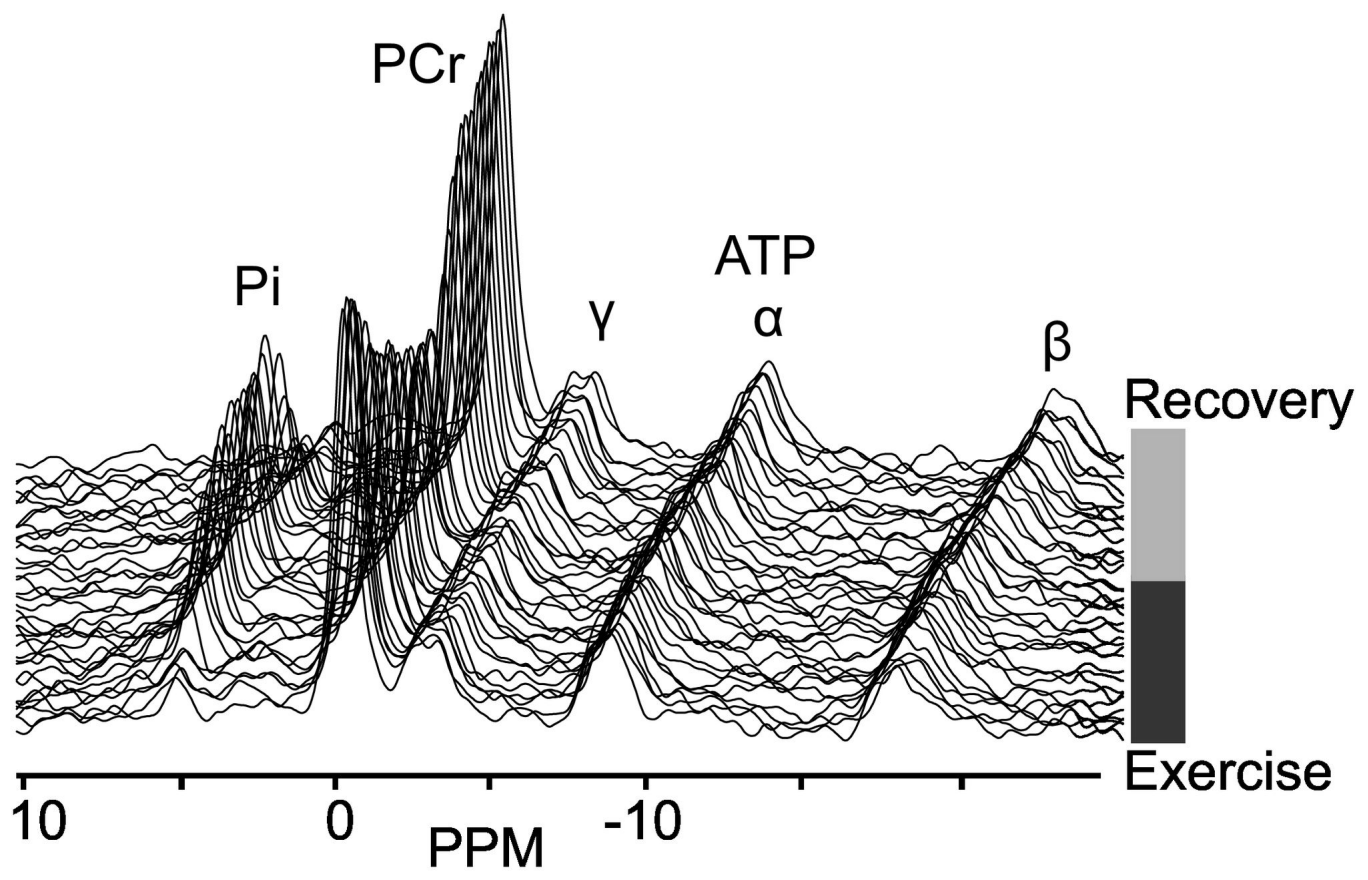
Dynamic series of ^31^P NMR spectra. Dynamic series of ^31^P NMR spectra obtained from the lateralis muscle during bicycle exercise and recovery. Peaks visible are inorganic phosphate (Pi), phosphocreatine (PCr) and three signals for adenosine triphosphate (γ-, α- and β-ATP).

**Figure 5:**
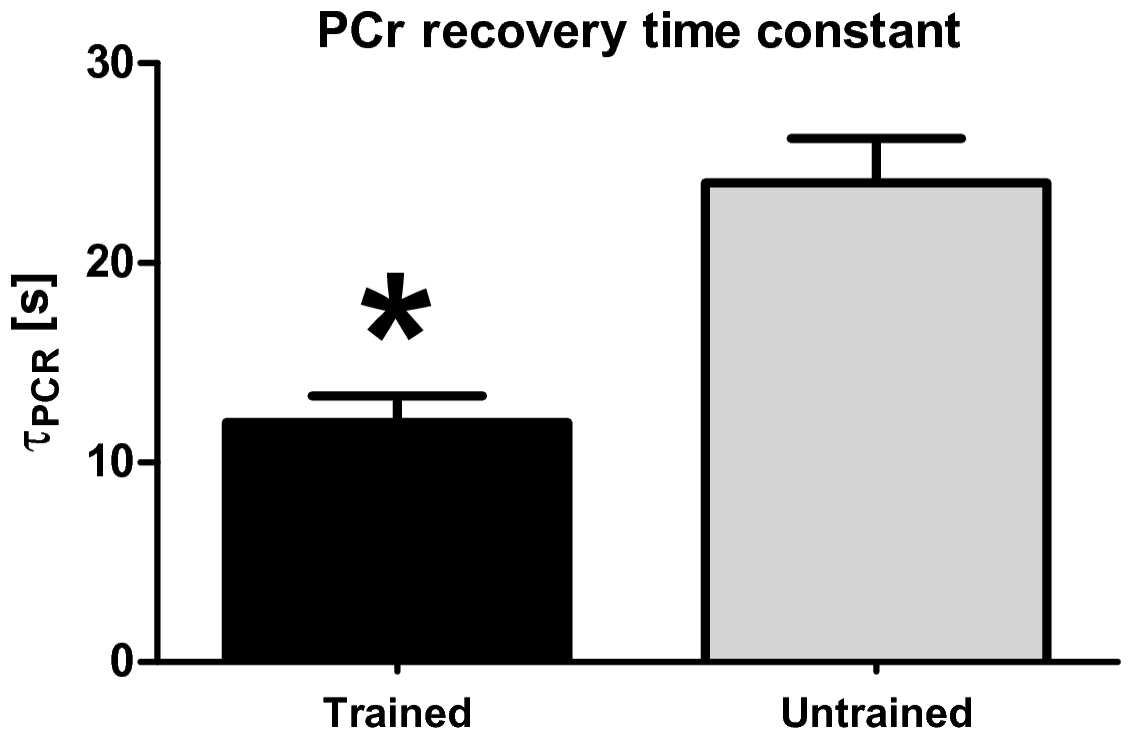
Bar plot of PCr recovery rate trained/untrained. Bar plot of PCr recovery rate with a significant faster τ_PCr_ in the endurance trained athletes (12 ± 3 s) compared to the normal physical active group (24 ± 5 s) (P < 0.05).

An example of PCr dynamics during exercise and recovery is shown in Figure 4. For the endurance trained athletes, a mean time constant τ_PCr_ of 12 ± 3 s (mean ± SD; n=5) was found, while in the normally active healthy subjects the mean value was significantly higher 24 ± 5s (mean ± SD; n=5) (Figure 5).

During and after exercise, tissue pH was determined based on the chemical shift between the Pi and PCr resonances. Average end-exercise pH was 6.9 ± 0.1 in all subjects of both groups. No difference was observed in Pi/ATP at rest between groups.

### Experimental observations compared to model predictions

**Figure 6:**
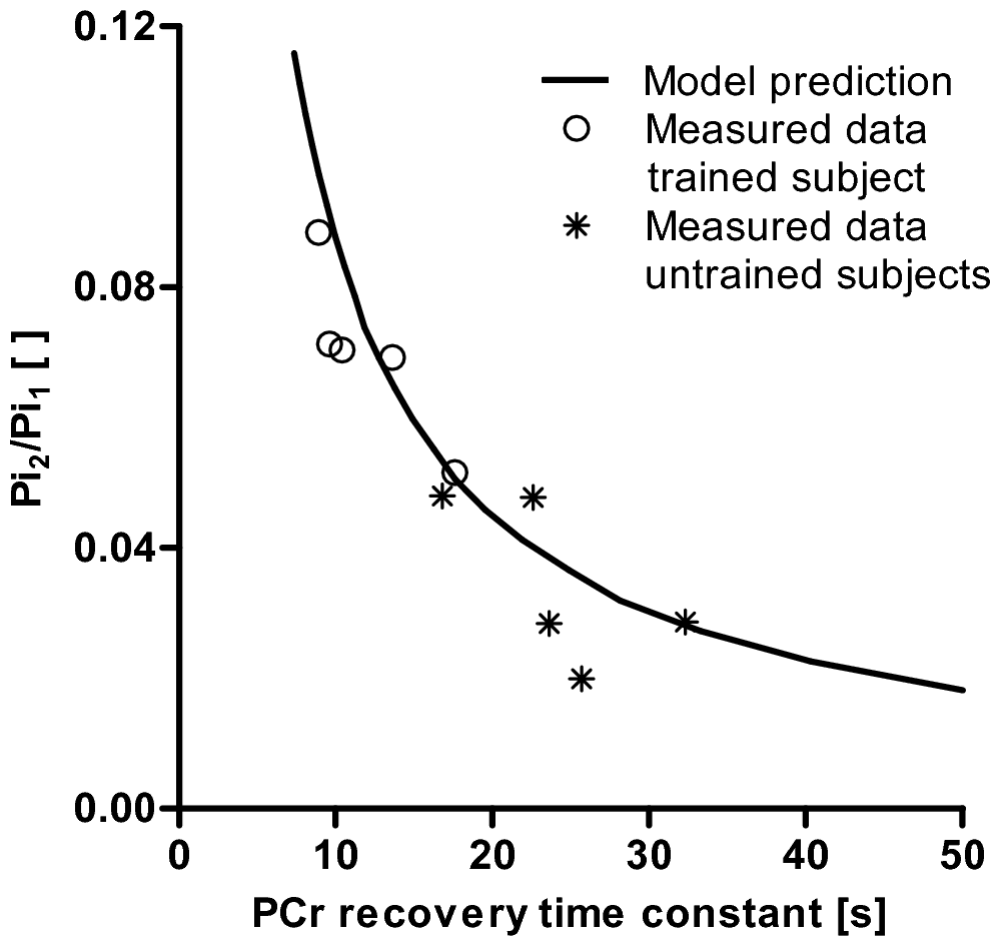
Model prediction of the relation between PCr recovery time constant and Pi_2_/Pi_1_. Model prediction of the relation between PCr recovery time constant and Pi_2_/Pi_1_. Experimental data points from the trained group are indicated by o, and from the untrained group with *.

Figure 6 shows the comparison of the empirically observed relation between τ_PCr_ and Pi_2_/Pi_1_ and the predicted relation (solid black line). Without any model parameter optimalization already an excellent agreement between the experimental data and model predictions was observed (R^2^ = 0.75). 

## Discussion

In this study, we investigated the relation between the Pi_2_/Pi_1_ ratio in resting quadriceps muscle and the PCr recovery rate following moderate exercise of the upper legs in healthy human subjects and trained athletes using a combination of experimental and numerical approaches. Specifically, the hypothesis was tested that the amplitude of the Pi_2_ signal in resting muscle reflects the mitochondrial density of the muscle under investigation. If confirmed, ^31^P MRS of resting human muscle at 7T may offer a novel, practical method for non-invasive assay of mitochondrial density and function in human muscle.

In agreement with a previous study [8], a Pi_2_ resonance was reproducibly detected 0.4 ppm downfield from the cytosolic Pi (Pi_1_) signal in both the endurance trained and the normal physically active subjects. A twofold higher Pi_2_/Pi_1_ ratio was found in the quadriceps muscle of athletes compared to normally active subjects, as well as a faster PCr recovery rate. Both aspects confirm a higher oxidative capacity of quadriceps muscle in these trained individuals and are in agreement with the reported magnitude of increase of mitochondrial density in biopsy studies as a result of endurance training [17]. No significant difference was observed in Pi/ATP in resting spectra between the two groups. Assuming that endurance training has no major effect on ATP concentration in the cytoplasm, the higher Pi_2_/Pi_1_ ratio was therefore attributed to an increase in Pi_2_ signal. Together, these results provides the first important new piece of evidence supporting the hypothesis that the Pi_2_ resonance in resting muscle is associated with the mitochondrial pool in the tissue

The PCr recovery rate after exercise was significantly faster in the endurance trained athletes confirming a higher oxidative capacity of quadriceps muscle in these trained individuals. Any interference of differences in muscle fiber type recruitment between the trained and untrained group on PCr recovery dynamics was assumed to be small since similar exercise intensity caused 50% of PCr depletion in all subjects. Any interference from variation in muscle sampling arising from localization differences between the static and dynamic measurements were minimized by placement of the surface coil at the same position, by the same person who performed the high field measurements.

The second important piece of evidence in support of the proposed hypothesis stems from the mechanistic computational modeling. Our results indicate that the observed relation between the Pi_2_/Pi_1_ and τ_PCr_ matched remarkable well (R^2^ = 0.75) with the theoretically expected relation taking into account the biochemical and biophysical properties of mitochondria. These results support the hypothesis that the Pi_2_/Pi_1_ ratio measured in resting skeletal muscle is closely related to mitochondrial density and therefore may provide a non-invasive biomarker of this important clinical parameter.

There is one concern regarding a strict mitochondrial origin of the Pi_2_ signal in resting muscle that warrants addressing, in particular in the context of the present study. As discussed previously [8] free Pi in blood vessels within the NMR-sampled muscle mass could potentially contribute to the measured Pi_2_ signal. Similar to the mitochondrial compartment, the pH in blood plasma is alkaline with a pH of 7.4 in resting humans [18]. Endurance training has been shown to cause an increase in capillary density of muscle [19]. The specific concern is therefore that the observed higher Pi_2_/Pi_1_ ratio in an endurance trained subject could in part be the result of increased capillary density of their leg muscle. However, the maximal increase in capillary density as a result of extreme endurance training is reported to be on the order of 20-40% [20]. In contrast, we found on average a 250% increase in normalized Pi_2_ signal in trained quadriceps muscle compared to controls (Figure 3). Therefore any bias in our conclusions caused by increased capillary density of trained muscle was only minor. In all cases the voxel analyzed was carefully positioned inside the muscle tissue, and did not include any visible major blood vessels. We have investigated if the amplitude of the Pi_2_ peak was significantly higher if we intentionally incluse visible vessels in the selected voxel. In most subjects however this was difficult, because the largest visible blood vessels are located further away from the surface coil, and therefore the S/N is not sufficient to separately observe the Pi_2_ peak. In the subjects in which the S/N was high enough in the area with visible vessels, there was no significant difference in Pi_2_ intensity with the chosen voxel in the lateralis muscle.

The ability to use ^31^P MRS in resting skeletal muscle at high field as a tool to provide information about mitochondrial properties, may benefit clinical investigations of mitochondrial function in human muscle. Changes in mitochondrial function have been associated with several disorders, including diabetes [21] and chronic heart failure [22]. *In vivo* mitochondrial capacity in these patients is quantified by the PCr recovery method [23]. In addition, often, muscle biopsy samples are obtained to determine whether a prolonged PCr recovery period is a result of a decreased number of mitochondria or intrinsic mitochondrial dysfunction. The Pi_2_/Pi_1_ read-out could provide similar information non-invasively; a decrease or even absence of any detectable Pi_2_/Pi_1_ resonances could point towards a reduced mitochondrial content, whereas no changes in Pi_2_/Pi_1_ ratio in combination with a prolonged PCr period could indicate the presence of actual intrinsic mitochondrial dysfunction. This information could also benefit the diagnosis of mitochondrial myopathies as well as follow-up of the efficacy of treatment strategies. This particular class of human mitochondrial disease is characterized by a heterogeneous phenotype, ranging from patients with severe mitochondrial dysfunction reflected by a dramatic prolongation of PCr recovery kinetics to patients with more or less normal PCr recovery kinetics [24]. The latter may be the result of a compensatory increase in the number of mitochondria, which should then be detectable by an increased Pi_2_/Pi_1_ ratio. Moreover, the non-invasive method for read-out of mitochondrial density proposed in the present study could be valuable in evaluating the effectiveness of stimulating mitochondrial biogenesis by e.g., exercise therapy.

A final promising aspect of the method is the fact that if the Pi_2_ resonance amplitude in muscle reports on mitochondrial matrix Pi content, the Pi_2_ resonance *frequency* reports on the pH gradient across the inner mitochondrial membrane (IMM). It has been well documented that mitochondria in resting muscle of patients with severe mitochondrial myopathy phenotypes cannot sustain a normal basal free energy potential for ATP hydrolysis [25]. While this suggests a significantly reduced proton motive force across the IMM in these patients, this corollary has in fact never been tested in vivo. Measurement of the Pi_2_ resonance frequency at 7T in muscle of these patients could provide this wanting information, as well as provide an additional read-out for diagnosis and therapy effect monitoring.

In summary, this study provides evidence that the Pi_2_/Pi_1_ ratio measured in skeletal muscle is closely related to mitochondrial content. Testing the added value of this biomarker in clinical investigation of mitochondrial diseases provides promising directions for future application of high-field *in vivo* NMR spectroscopy.
